# Structural Mechanisms Driving the Selective Efficacy of Oxamniquine against *Schistosoma mansoni* and *Schistosoma japonicum*

**DOI:** 10.1007/s12013-025-01756-9

**Published:** 2025-04-19

**Authors:** Kehinde F. Paul-Odeniran, Emmanuel A. Iwuchukwu, Paul O. Odeniran

**Affiliations:** 1 Centre for Biomolecular Sciences, School of Pharmacy, University of Nottingham, University Park, Nottingham, UK; 2 Department of Natural Sciences, Faculty of Pure and Applied Sciences, Precious Cornerstone University, Ibadan, Oyo State Nigeria; 3 Protein Structure-Function and Research Unit, School of Molecular and Cell Biology, Faculty of Science, University of the Witwatersrand, Braamfontein, Johannesburg, South Africa; 4 Department of Veterinary Parasitology and Entomology, Faculty of Veterinary Medicine, University of Ibadan, Ibadan, Nigeria

**Keywords:** Oxamniquine, Schistosoma, Binding free energy, Structural mutation

## Abstract

Oxamniquine (OXA) exhibits selective efficacy against different *Schistosoma* species, with the highest activity observed in *Schistosoma mansoni* sulfotransferase (*Sm*SULT) and the lowest in *Schistosoma japonicum* sulfotransferase (*Sj*SULT). This study utilises advanced atomistic and molecular simulations to elucidate the structural dynamics induced by OXA binding to *Sm*SULT and *Sj*SULT, aiming to unravel the underpinnings of this selective efficacy. Binding free energy (BFE) analyses revealed a markedly higher affinity of OXA for *Sm*SULT (−48.04 kcal/mol) compared to wt*Sj*SULT (−22.84 kcal/mol), with a significant restoration of binding affinity (−39.23 kcal/mol) observed in *Sj*SULT following the mutation of Val139 to Gly139. Comprehensive conformational assessments highlighted that *Sm*SULT-OXA achieves its superior efficacy by stabilising the protein structure, in stark contrast to the erratic conformational behaviour of wild-type *Sj*SULT. Notably, this erratic behaviour is ameliorated upon mutation, leading to a restoration of OXA’s efficacy in *Sj*SULT. These insights elucidate the structural mechanisms underpinning OXA’s selective efficacy and provide valuable perspectives on its targeted action against *Schistosoma spp*.

## Introduction

Human schistosomiasis poses a significant public health challenge caused by the blood fluke *Schistosoma* spp. As of 2021, the World Health Organization reported that approximately 251 million individuals required preventive chemotherapeutic intervention, with over 700 million residing in approximately 78 endemic countries [1]. Additionally, 280,000 deaths are attributed to this disease annually [2]. Sub-Saharan Africa bears the brunt of this global burden, accounting for 90% of the cases [3]. Human beings are infected by three primary species of schistosomes: *Schistosoma haematobium*, *Schistosoma mansoni*, and *Schistosoma japonicum*. *S. haematobium* and *S. mansoni* are prevalent in Africa and the Middle East, while *S. mansoni* exclusively exists in the Americas [4–6]. *S. japonicum* is localised mainly in the Asian region [7].

*Schistosoma* has a two-host lifecycle involving freshwater snails as the intermediate host and mammals as the final host [8]. After infiltrating snails, the eggs develop into miracidia, then sporocysts, which multiply into cercariogenous or sporocystogenous sporocysts. Snails release these into water. Humans become infected through water contact; cercariae penetrate the skin, transforming into schistosomula, which travel to body tissues [8]. *S. haematobium* settles in the bladder, rectum, and ureters; *S. mansoni* in the large or small intestine; and *S. japonicum* in the small intestine [9]. Schistosomula mature into schistosomes and adult worms. Infected humans contribute to the continuation of the cycle by releasing *Schistosoma* eggs into freshwater bodies through parasite-containing faeces and urine. These eggs hatch, releasing miracidia, which are then taken up by freshwater snails, thus perpetuating the life cycle of *Schistosoma* [10].

Schistosomiasis presents with initial symptoms like an itchy skin rash upon water contact. In the acute phase, individuals may experience fever, fatigue, muscle aches, and abdominal pain [11]. As the disease progresses, organ-specific manifestations emerge, such as blood in urine, painful urination, and abdominal discomfort for *S. haematobium*, or diarrhoea and liver enlargement for *S. mansoni* and *S. japonicum* [12]. If left untreated, chronic infections can lead to severe complications, including bladder and kidney damage or liver fibrosis [13]. Praziquantel stands as the primary chemotherapeutic intervention for schistosomiasis, demonstrating efficacy against the three major *Schistosoma spp*. Reported cure rates range between 60 and 90% [14]. While predominantly effective against adult worms, its resistance has been documented primarily due to widespread monotherapy usage [12]. Before praziquantel, oxamniquine (OXA), which is a prodrug, was the leading anti-schistosomal drug. However, due to emerging resistance and its species-specific activity, as it was only effective against *Schistosoma mansoni*, praziquantel gradually replaced it, driven in part by its lower production cost [15, 16].

The validated catalytic enzyme for OXA activation is PAPS-dependent Sulfotransferase, which is specifically present in the three primary *Schistosoma* species affecting humans [17]. OXA binds to this enzyme, initiating its activation through sulfation and consequently leading to its schistosomicidal effect [17]. Notably, the three *Schistosoma* species exhibit variations in their sequence identity, and the species-specific response to OXA is an area of active investigation [18]. Understanding the mechanism of this selective inhibition prompted a comprehensive study by Taylor et al. who delved into the enzyme kinetics of *Schistosoma* SULT in complex with OXA. Kinetic analyses revealed that *Sm*SULT displayed the highest enzymatic activity with OXA as the substrate, as indicated by the kcat/KM values, followed by *Schistosoma haematobium* and the least by *Schistosoma japonicum* [19]. Additionally, the research shed light on the stereoisomer of OXA that preferentially binds to specific SULT *Schistosoma* variants [19].

In another study, Rugel et al. conducted experiments involving the introduction of mutations in the amino acid residues of *Schistosoma* SULTs that come into contact with OXA. Their aim was to investigate the role of these mutations in either hindering or facilitating the activation of OXA [18]. A noteworthy finding of their study was the re-establishment of OXA activation when Val139 in *Sj*SULT was mutated to Gly139. This suggests a correlation between the amino acid residues within the binding site of SULT and the potential inactivation of OXA [18]. Additionally, the research shed light on the stereoisomer of OXA that preferentially binds to specific SULT *Schistosoma* variants [19].

Building on these findings, we selected *S. mansoni* and *S. japonicum* for this comparative study based on their contrasting enzymatic profiles. *Sm*SULT exhibits the highest OXA activation efficiency, while *Sj*SULT demonstrates the weakest response—unless Val139 is mutated to Gly139. *S. haematobium*, which shows intermediate activity, was excluded to enable a clearer, mechanistically distinct comparison between the two extremes of OXA responsiveness. This binary contrast enhances our ability to evaluate residue-specific interactions, conformational shifts, and binding energetics. Moving beyond static structural insights, we employed atomistic and molecular simulations to investigate the dynamic mechanisms underlying OXA’s species-specific efficacy. We further explored how the Val139→Gly139 mutation in *Sj*SULT restores OXA activity, providing critical insights into species-selective drug responsiveness. By bridging computational simulations with experimental observations, our study addresses a crucial gap in understanding OXA’s mechanism of action and offers a foundation for rational design of next-generation derivatives with broader efficacy and reduced resistance potential.

## Methodology

### Sequence Alignment and Preparations of Systems

The sequence alignment of *Sm*SULT (Uniprot: G4VLE5) and *Sj*SULT (Uniprot: C1LER5) was conducted using Clustal Omega [20–22]. This tool facilitates the identification of conserved and variable regions by aligning sequences based on similarity, aiding in the analysis of specific residues that may explain OXA’s selective efficacy against these *Schistosoma* species.

**Fig. 1:**
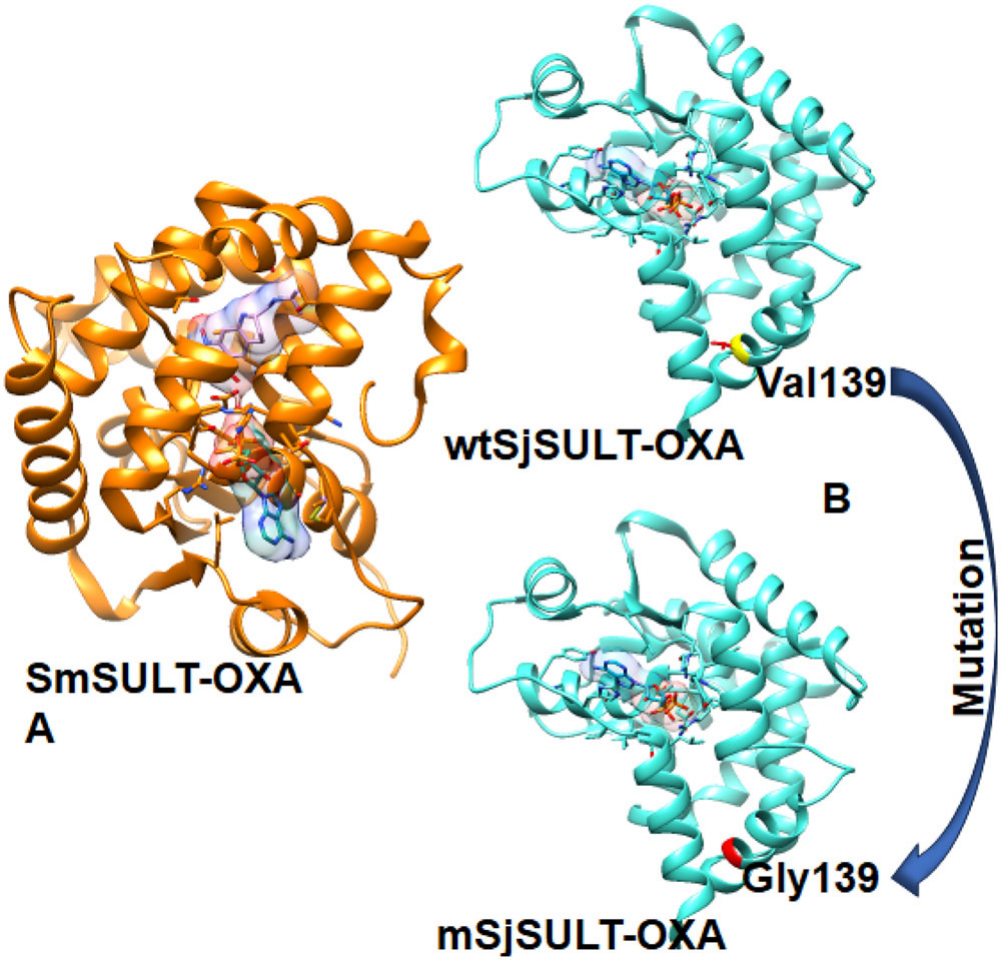
A Structural Representation of Ligand-bound *Sm*SULT and *Sj*SULT. **A** shows *Sm*SULT in a bound state with OXA and the cofactor whereas **B** indicates the point mutation in wt*Sj*SULT wt*Sj*SULT-OXA to m*Sj*SULT-OXA

Taylor et al. had earlier determined the enantiomer of OXA that preferentially binds to the different *Schistosoma* spp. *Sj*SULT prefers the R-OXA, while *Sm*SULT has no preference and can bind to either of the OXA enantiomers [19]. The *Sm*SULT/S-OXA complex was downloaded from the Protein Data Bank (PDB code: 5BYK) [19], along with *Sj*SULT (PDB code: 5TIZ) [19]. The R-OXA in the *Sh*SULT/R-OXA complex (PDB code: 5TIX) was superimposed on *Sj*SULT to generate the *Sj*SULT/R-OXA complex (Taylor et al., 2017). For mutational studies in *Sj*SULT, the Val-139-Gly mutation was created using UCSF Chimera (Fig. 1) [23–26]. The systems were then prepared for molecular dynamics simulation, including the apo form (protein bound to adenosine-3′–5′-diphosphate) where we have *Sm*SULT, wild-type *Sj*SULT (wt*Sj*SULT) and the complexes (protein bound to adenosine-3′–5′-diphosphate and the respective OXA enantiomer) where we *Sm*SULT-OXA, wt*Sj*SULT-OXA and mutant *Sj*SULT (m*Sj*SULT).

### Preparation of the Systems for Molecular Dynamics Simulation

All protein-ligand structures were first visually inspected and validated using UCSF Chimera to ensure there were no missing atoms, residues, or structural loops. Following the point mutation (Val139→Gly139) introduced in *Sj*SULT, the mutated structure was subjected to an initial energy minimisation to relax local steric strain and ensure structural integrity. All systems—including *Sm*SULT, wt*Sj*SULT, and m*Sj*SULT—underwent two-stage energy minimisation consisting of 2500 steps of steepest descent followed by 5000 steps of conjugate gradient minimisation. The minimised complexes were then solvated in an octahedral box of TIP3P water molecules with a 10 Å buffer distance from the protein surface. Counterions (Na^+^ or Cl^−^) were added to neutralise the system. The ff14SB force field was used to parameterise the proteins, and ligand parameters were generated using the GAFF2 force field via the ANTECHAMBER module. Each system was gradually heated from 0–300 K over 50 ps using a constant volume ensemble (NVT), applying a Langevin thermostat with a collision frequency of 1 ps^−1^. This was followed by a 1 ns equilibration under constant pressure (NPT) conditions using a Berendsen barostat with a pressure relaxation time of 1 ps. A positional restraint of 5 kcal/mol·Å^2^ was applied to solute atoms during the heating phase and was removed during equilibration. Subsequently, production molecular dynamics (MD) simulations were performed for 300 ns under NPT conditions with periodic boundary conditions and particle mesh Ewald electrostatics. Each system was simulated in duplicate using independently generated initial velocities to ensure reproducibility of the results and to capture potential variability in conformational behaviour.

### Post- Molecular Dynamics Simulation Analysis

The analysis of the MD-generated trajectories was carried out using the CPPTRAJ and PTRAJ modules, with data visualised using the Origin software [27, 28]. Post-MD structural and conformational evaluations were carried out. Binding free energy calculation using the Molecular Mechanics/Poisson-Boltzmann Surface Area (MM/PBSA) approach [29, 30]. Visualisation and further analyses were conducted using Molegro Molecular Viewer and UCSF Chimera software [23].

### Thermodynamics Calculations

The binding free energy of OXA to the binding site of *Sm*SULT and wt*Sj*SULT and m*Sj*SULT was estimated via the Molecular Mechanics Generalised Born and Surface Area (MM/GBSA) method. This method, a robust tool for assessing ligand interactions with biological macromolecules, was implemented using the MM/GBSA.py script integrated into AmberTools18. To ensure thorough sampling, the binding free energy was calculated using the full 300 ns molecular dynamics trajectory for each system with snapshots extracted at 1 ns intervals (300 frames per complex). The binding free energy (ΔG_bind) is calculated as follows:

1 $${\Delta {\rm{G}}}_{{\rm{bind}}}={{\rm{G}}}_{{\rm{complex}}}-({{\rm{G}}}_{{\rm{receptor}}}+{{\rm{G}}}_{{\rm{inhibitor}}})$$

2 $${\Delta {\rm{G}}}_{{\rm{bind}}}={\Delta {\rm{G}}}_{{\rm{gas}}}+{\Delta {\rm{G}}}_{{\rm{sol}}}-{\rm{T}}\Delta {\rm{S}}$$

3 $${\Delta {\rm{G}}}_{{\rm{gas}}}={\Delta {\rm{E}}}_{\mathrm{int}}+{\Delta {\rm{E}}}_{{\rm{ele}}}+{\Delta {\rm{E}}}_{{\rm{vdW}}}$$

4 $${\Delta {\rm{G}}}_{{\rm{sol}}}={\Delta {\rm{G}}}_{{\rm{PB}}}+{\Delta {\rm{G}}}_{{\rm{np}},{\rm{sol}}}$$

The interactions contributing to gas-phase energy (ΔG_gas_) include internal forces (ΔE_int_), electrostatic interactions (ΔE_ele_), and van der Waals forces (ΔE_vdW_) [31]. Meanwhile, solvation-free energy (ΔG_sol_) arises from both polar (ΔG_PB_) and non-polar (ΔG_np,sol_) solvation contributions. To further understand binding free energy (ΔG_bind_), an analysis was conducted, breaking it down into the energy contributions of individual residues at the binding site [32]. This detailed per-residue energy decomposition (PRED) was refined by integrating the binding residues of certain natural compounds into the MMPB/SA.py script from AmberTools18. Such an approach was pivotal in identifying key residues within *Schistosoma* SULT that influence ligand binding affinity and stability.

## Results and Discussion

### Comparative Sequence Alignment of *Sm*SULT and *Sj*SULT: Insights into Residue Disparity

**Fig. 2:**
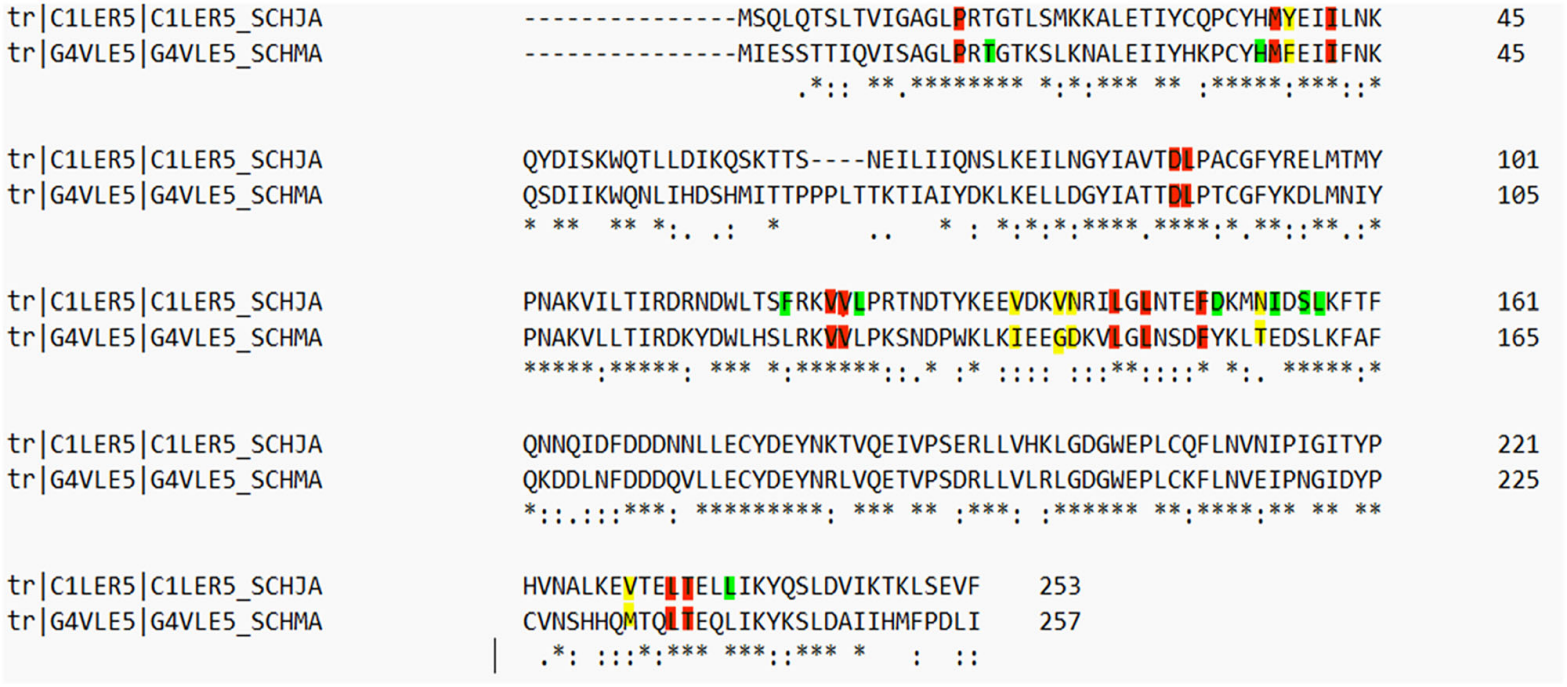
Sequence Alignment of *Sm*SULT and *Sj*SULT, Highlighting Conserved and Variable Regions. The asterisks represent conserved regions in the complete protein sequences of both *S. japonicum* and *S. mansoni*. Red indicates conserved binding site residues, yellow indicates non-conserved binding site residues, and green highlights other binding site residues that interact with OXA specific to each protein

Figure 2 shows the detailed sequence alignment between *Sm*SULT and *Sj*SULT. This alignment is crucial for understanding their evolutionary relationship, structural conservation, divergence, and functional similarities [33]. Most importantly, it may help us understand the disparity in the efficacy of OXA in *Sj*SULT and *Sm*SULT, as Taylor et al. suggested that differences in active site residues in both proteins may be responsible for the observed disparity [19]. The overall sequence similarity is 59.29%. The intersecting residues in *Sm*SULT/*Sj*SULT are PRO16/PRO16, MET38/MET38, ILE42/ILE42, ASP91/ASP87, LEU92/LEU88, VAL127/VAL123, VAL128/VAL124, LEU147/LEU143, PHE153/PHE149, LEU236/LEU232, THR237/THR233, and LEU147/LEU143. The differing residues are PHE39/TYR34, ILE140/VAL136, GLY143/VAL139, ASP144/ASN140, THR157/ASN153, and MET233/VAL224 respectively. Subsequent analyses reveal the impact of these residues on the structural and conformational properties of the proteins, contributing to the observed disparity in the efficacy of OXA in both SULTs. The structural differences, particularly in the active site regions, may influence the binding affinity and stability of OXA, thereby affecting its overall efficacy.

### Binding Free Energy Analysis of OXA-bound *Sm*SULT and *Sj*SULT

We estimated the binding free energy (BFE) values after a 300 ns MD simulation, carefully accounting for entropic effects. This detailed analysis validated the experimental findings, reinforcing that OXA is indeed more effective in *Sm*SULT than in wt*Sj*SULT. Specifically, the binding affinity of OXA to *Sm*SULT was significantly higher, with a BFE value of −48.04 kcal/mol, compared to −22.84 kcal/mol for its binding to wt*Sj*SULT. Moreover, when Val139 in wt*Sj*SULT was mutated to Gly139, the efficacy of OXA in *Sj*SULT was remarkably restored, as evidenced by the improved BFE value of −39.23 kcal/mol. This mutation appears to facilitate a binding affinity more akin to that observed in *Sm*SULT, further substantiating the experimental results.

To gain deeper insights into the effect of the residue mutation, we investigated the contributions of various energy components to the endpoint BFE values of wt*Sj*SULT-OXA and m*Sj*SULT-OXA (Table 1). Despite the lower energy cost of solvation (∆G(GB)) of OXA in wt*Sj*SULT (−61.61 kcal/mol), its binding free energy was less favourable than that of OXA in m*Sj*SULT (150.59 kcal/mol). However, the high energy cost of solvation in the latter was compensated for by the dense electrostatic interaction of OXA in m*Sj*SULT (−144.23 kcal/mol) compared to wt*Sj*SULT, where electrostatic interactions were almost non-existent (77.06 kcal/mol). The electrostatic bond is regarded as the strongest bond between a ligand and the protein, and alterations in this interaction will affect the binding free energy of the ligand to the protein [34]. The van der Waals interaction was also quite significant in m*Sj*SULT-OXA (−40.02 kcal/mol) compared to wt*Sj*SULT (−33.6 kcal/mol) (Table 1).

**Table 1:** Energy Components (kcal/mol) for the Interaction of OXA with *Sm*SULT, wt*Sj*SULT, and m*Sj*SULT Complexes

	Energy Components (kcal/mol)						
Complexes	∆E_vdW_	∆E_ele_	∆G_gas_	∆G_ele,sol(GB)_	∆G_np,sol_	∆G_sol_	∆G_bind_
*Sm*SULT-OXA	−39.83 ± 0.13	−294.97 ± 0.49	−334.41 ± 0.48	292.27 ± 0.42	−5.90 ± 0.098	286.37 ± 0.41	−48.04 ± 0.17
wt*Sj*SULT−OXA	−33.6 ± 0.15	77.06 ± 0.84	43.47 ± 0.85	−61.61 ± 0.8	−4.7 ± 0.02	−66.31 ± 0.79	−22.84 ± 0.23
m*Sj*SULT−OXA	−40.02 ± 0.16	−144.23 ± 0.54	−184.26 ± 0.56	150.49 ± 0.54	−5.47 ± 0.01	145.02 ± 0.53	−39.23 ± 0.15

The orientation of OXA within the binding sites of wt*Sj*SULT and m*Sj*SULT, as interpreted from the non-polar solvation energy (ΔG_np,sol_), appears to have had a significant impact on the overall binding energy. Specifically, OXA in wt*Sj*SULT showed the lowest non-polar solvation energy at −4.7 kcal/mol, implying that the ligand was more exposed to the surface, increasing its interaction with the polar environment. This orientation likely resulted in a lower total binding free energy compared to OXA in m*Sj*SULT and *Sm*SULT, which exhibited non-polar solvation energies of −5.47 and −5.9 kcal/mol, respectively.

**Fig. 3:**
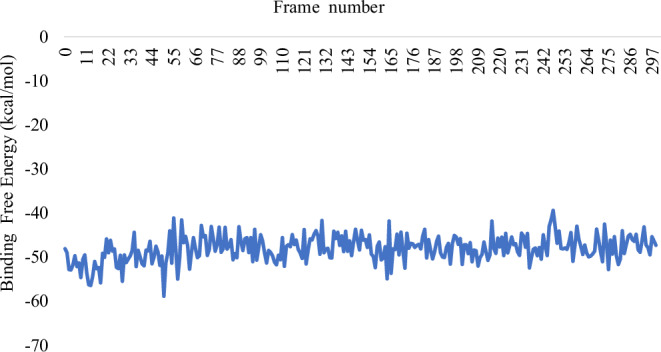
Binding Free energy of *Sm*SULT-OXA complex sampled over the entire Simulation Course

**Fig. 4:**
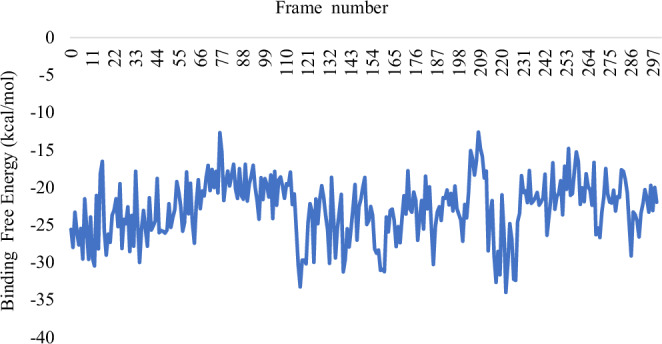
Binding Free energy of wt*Sj*SULT-OXA complex sampled over the entire Simulation Course

**Fig. 5:**
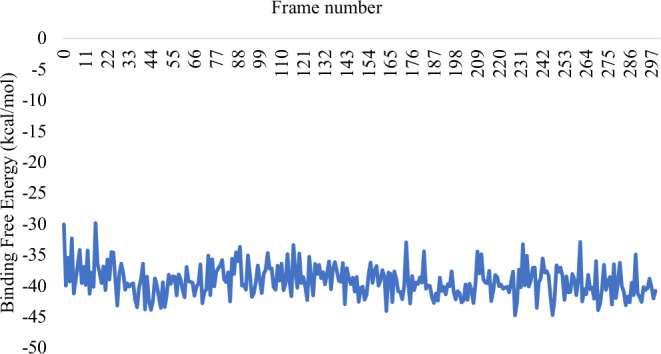
Binding Free energy of m*Sj*SULT-OXA complex sampled over the entire Simulation Course

The comparative analysis of the BFE graphs in Figs. 3, 4, and 5 reveals that the *Sm*SULT-OXA complex exhibits the highest and most consistent binding affinity, followed by the m*Sj*SULT-OXA complex. In contrast, the wt*Sj*SULT-OXA complex shows notably weaker binding, characterised by fluctuating BFE values indicative of reduced stability and consistency. This suggests that its binding affinity is neither as robust nor as stable as that observed in the *Sm*SULT complex. The mutation from Val139 to Gly139 in *Sj*SULT significantly enhances the binding affinity and stability of the OXA complex, aligning its characteristics more closely with those of the *Sm*SULT complex. The energetic profiles remained consistent across replica simulations, further validating the reproducibility of the observed binding trends (see Supplementary Table 1).

### Comprehensive Per-residue Energy Decomposition for OXA Binding in *Sm*SULT, wt*Sj*SULT and m*Sj*SULT

Our study meticulously analysed the energy distribution per residue within the binding sites, aiming to identify essential residues crucial for OXA binding across the three proteins. Initially, we compared the contributions of intersecting residues in *Sm*SULT and wt*Sj*SULT. Subsequently, our focus shifted to analysing residue-specific binding energies in both wild-type and mutant *Sj*SULT proteins, providing deep insights into how mutations affect the stability and affinity of the protein-ligand complex.

**Fig. 6:**
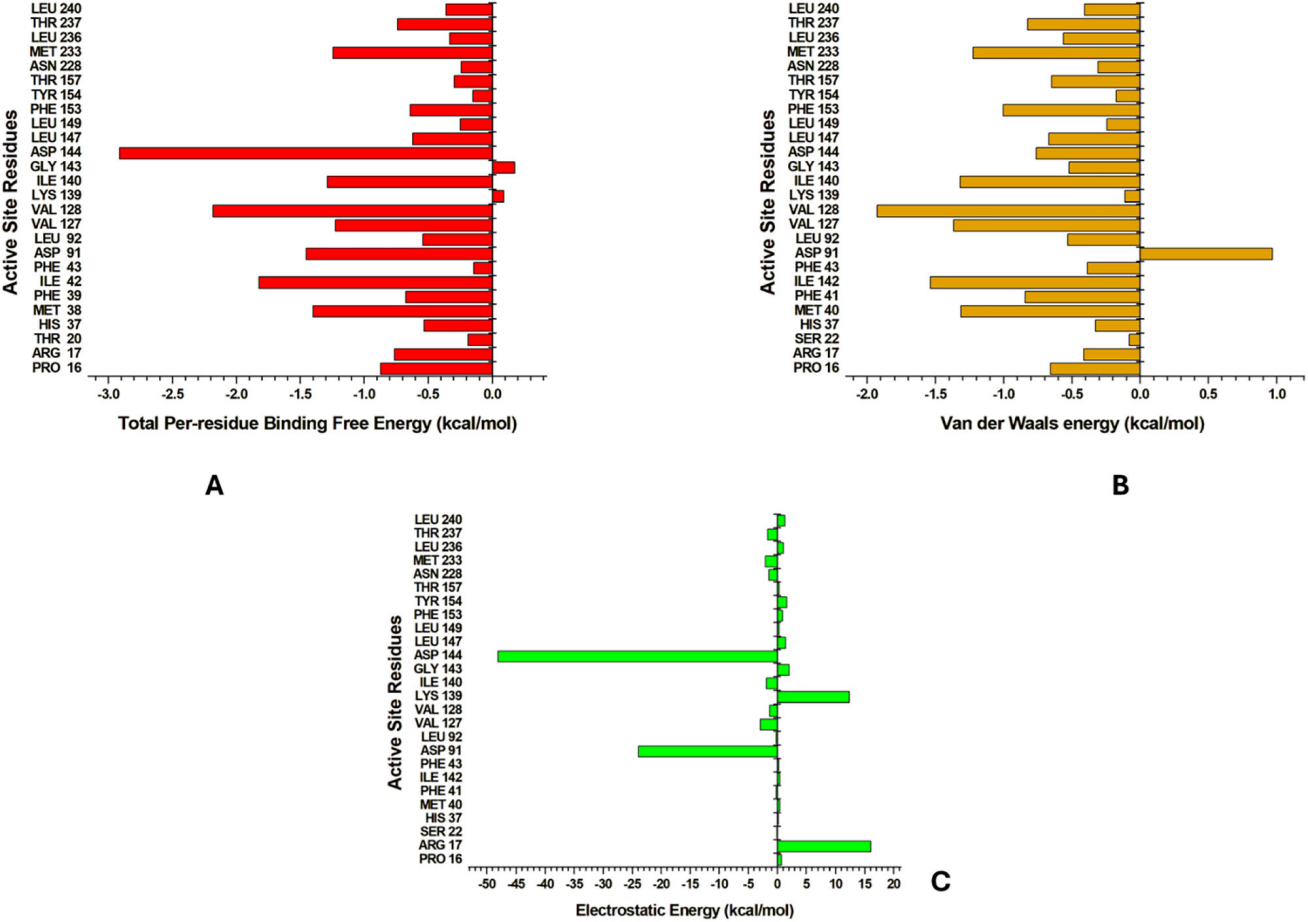
Energy Contributions of the Binding Site Residues of *Sm*SULT to the Binding Free Energy of OXA. **A** Total per-residue binding energy, **B** Van der Waals contribution, and **C** Electrostatic energy contribution

**Fig. 7:**
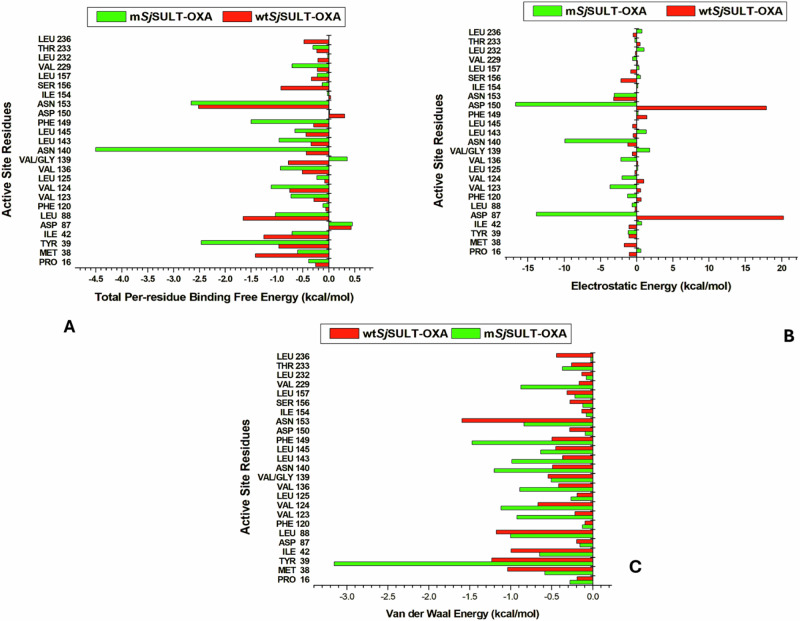
Energy Contributions of the Binding Site Residues of wt*Sj*SULT and *mSj*SULT to the Binding Free Energy of OXA. **A** Total per-residue binding energy, **B** Van der Waals contribution, and **C** Electrostatic energy contribution

Several residues emerged as significant contributors, each demonstrating substantial impacts with binding energies equal to or greater than −1 kcal/mol. In both *Sm*SULT and wt*Sj*SULT (Figs. 6A and 7A), PRO16 exhibited stronger binding energy (−0.87 kcal/mol) in *Sm*SULT compared to wt*Sj*SULT (−0.247 kcal/mol), indicating a more stable interaction. MET38 showed similar contributions in both proteins, with −1.399 kcal/mol in *Sm*SULT and −1.413 kcal/mol in wt*Sj*SULT. ILE42 (−1.823 kcal/mol) and ASP91 (−1.453 kcal/mol) displayed stronger stabilising effects in *Sm*SULT compared to ILE42 (−1.252 kcal/mol) and ASP87 (0.434 kcal/mol) in wt*Sj*SULT. VAL127 (−1.224 kcal/mol) and VAL128 (−2.182 kcal/mol) also demonstrated significantly higher binding energy contributions in *Sm*SULT than VAL123 (−0.281 kcal/mol) and VAL124 (−0.752 kcal/mol) in wt*Sj*SULT. LEU147 (−0.62 kcal/mol) and PHE153 (−0.639 kcal/mol) showed slightly stronger interactions in *Sm*SULT compared to LEU143 (−0.338 kcal/mol) and PHE149 (−0.286 kcal/mol) in wt*Sj*SULT. These findings underscore the critical roles of residues such as PRO16, ILE42, ASP91, VAL127, and VAL128 in enhancing the binding stability of the OXA complex in *Sm*SULT compared to wt*Sj*SULT, highlighting their importance in complex stability and explaining why *Sm*SULT exhibits better binding free energy than wt*Sj*SULT.

In comparing wild-type and mutant *Sj*SULT, Franco et al. established the importance of ASN140 and ASN153 in OXA binding [19]. ASN140 exhibited a remarkable decrease in binding energy from −0.428 kcal/mol in wt*Sj*SULT to −4.499 kcal/mol in m*Sj*SULT-OXA, underscoring its pivotal role in maintaining complex stability, particularly in the mutant form. TYR39 also showed a significant decrease in binding energy, shifting from −0.958 kcal/mol in wt*Sj*SULT to −2.46 kcal/mol in m*Sj*SULT-OXA (Fig. 7A), emphasising its substantial contribution to complex stability. ASN153 demonstrated consistent contributions between the wild-type and mutant proteins. Additionally, GLY139 in the mutated form did not contribute positively to the total binding energy (0.359 kcal/mol), in contrast to VAL139 in the wild-type protein (−0.774 kcal/mol). This trend was similarly observed with ILE42 and LEU88, which significantly stabilised OXA in wt*Sj*SULT with binding energies of −1.252 and −1.65 kcal/mol, respectively. However, due to the mutation in *Sj*SULT, their contributions decreased to −0.702 and −1.028 kcal/mol, respectively, indicating notable structural or energetic changes resulting from the mutation. The electrostatic and Van der Waal energy contributions of each binding site residue to the total binding free energy are highlighted in Fig. 7B, C.

### Dynamic Time-resolved Interactions of OXA with *Sm*SULT and *Sj*SULT Variants

**Fig. 8:**
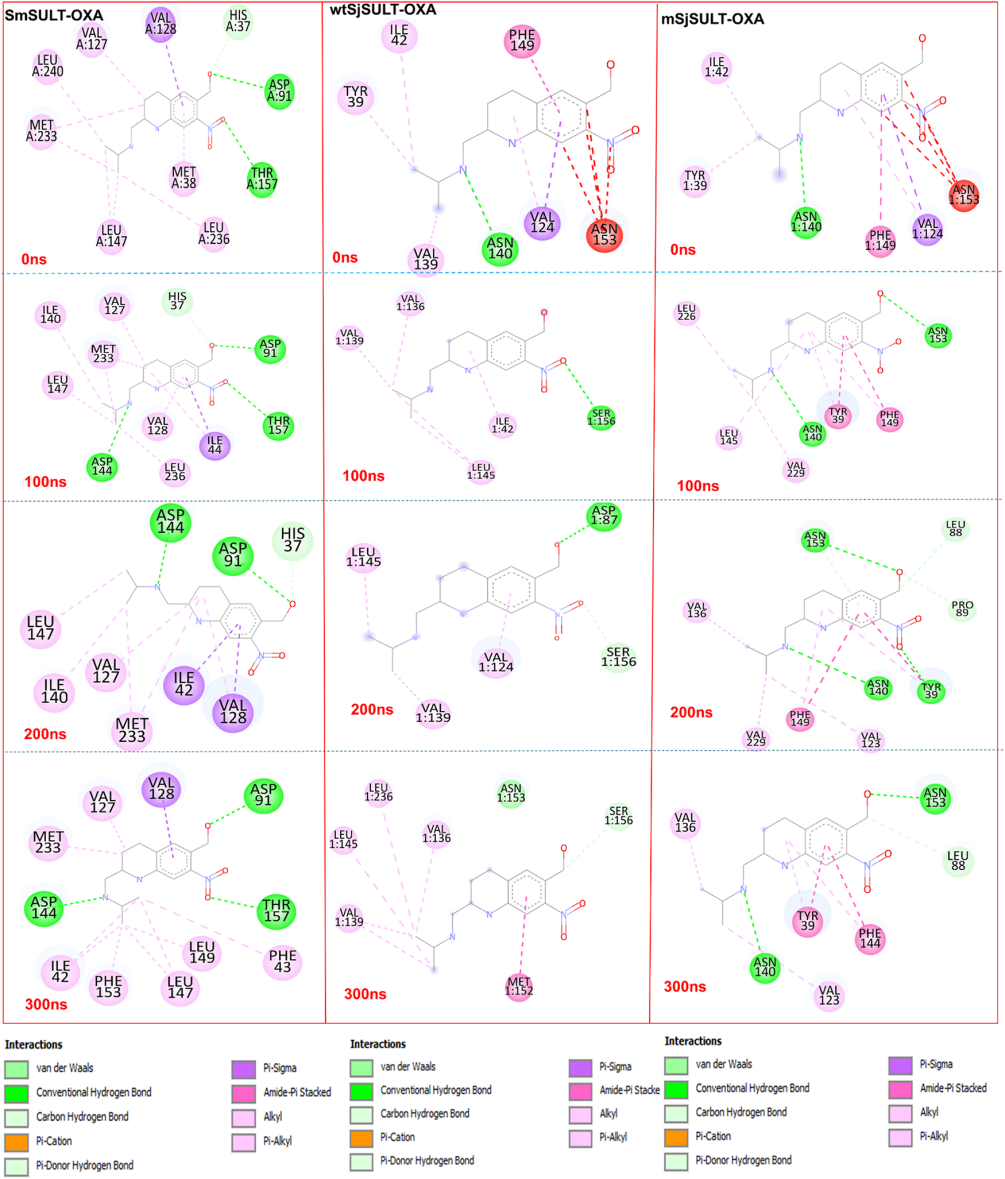
Molecular interaction maps showing the binding interactions of OXA with *Sm*SULT, wt*Sj*SULT, and m*Sj*SULT at different time points (0, 100, 200, and 300 ns)

In *Sm*SULT-OXA, ASP 144 and ASP 91 form strong electrostatic interactions primarily through hydrogen bonds, which significantly contribute to the stability of the ligand (Fig. 8). Van der Waals interactions involve VAL 127, VAL 128, LEU 147, LEU 236, and LEU 240, which are crucial for maintaining a hydrophobic environment [35]. The consistent hydrogen bonding by ASP 144 and ASP 91 throughout all stages ensures strong electrostatic attraction. Similarly, persistent van der Waals interactions by valine and leucine residues provide additional stabilisation. These stable and extensive interactions contribute to the highly favourable binding free energy of *Sm*SULT-OXA, reflected in its lowest ∆G_bind_ value among the complexes, indicating robust and efficient binding. In wt*Sj*SULT-OXA, interactions are predominantly driven by van der Waals forces, which are generally weaker chemical interactions [36]. This suggests a less stable ligand configuration and less favourable binding free energy over time. Conversely, the m*Sj*SULT-OXA complex displays stable and consistent interactions similar to *Sm*SULT-OXA, leading to favourable binding energy. It exhibits a combination of van der Waals and moderate electrostatic interactions, characterised by persistent hydrogen bonds and van der Waals interactions, contributing to its stability.

Focusing on key residues such as ASN 153 and ASN 140, these additional active site residues are believed to enhance catalytic efficiency in the two *Sj*SULT variants [19]. Their equivalents in *Sm*SULT, ASP 144 and THR 157, play significant roles as well. ASP 144 in *Sm*SULT consistently formed a conventional hydrogen bond with the ligand from 100 ns onwards, indicating its crucial role in maintaining ligand stability and orientation in the active site (Fig. 8). This interaction also accounts for its strong electrostatic energy contribution (−50 kcal/mol) to the overall binding free energy (Fig. 6C). In contrast, post-simulation snapshots showed that ASN 140 in wt*Sj*SULT did not visibly contribute to ligand stability in the binding site. However, in the mutant *Sj*SULT, ASN 140 consistently formed a conventional hydrogen bond with the ligand throughout the simulation, stabilising the ligand. This interaction may explain why OXA in the mutant *Sj*SULT regained its potency.

THR 157 in *Sm*SULT-OXA participates in hydrogen bonding with the ligand at 100 and 300 ns, suggesting its dynamic role in ligand binding by providing initial stability through hydrogen bonding. Similarly, in m*Sj*SULT-OXA, its ASN 153 counterpart forms multiple hydrogen bonds with the ligand at all time points, contributing to a stable binding configuration. The persistent hydrogen bonding by THR 157/ASN 153 indicates its importance in ensuring the ligand remains securely bound within the protein, allowing efficient catalysis in both *Sm*SULT and m*Sj*SULT. However, in wt*Sj*SULT, the ASN 153 interaction was absent throughout the simulation, which may explain its weaker binding free energy. The mutation in *Sj*SULT made the protein function almost exactly like *Sm*SULT in terms of binding and catalytic efficiency.

In the same vein, the analysis of hydrogen bond interactions between wt*Sj*SULT and m*Sj*SULT with OXA reveals key differences that explain the increased potency of OXA in m*Sj*SULT (Table 2). Notably, m*Sj*SULT shows higher H-bond occupancy, such as ASN140-OD1 with LIGAND-N3 (9.35%) and ASN153-OD1 with LIGAND-O1 (9.31%), indicating more stable interactions. In contrast, wt*Sj*SULT exhibits lower occupancy for ASN140-OD1 with LIGAND-N3 (2.60%) and ASN153-OD1 with LIGAND-O1 (2.57%), reflecting weaker binding. These enhanced bonds in m*Sj*SULT likely reduce ligand dissociation, contributing to greater binding efficiency. The mutations in m*Sj*SULT also refine the H-bond geometry, creating a more favourable binding environment for OXA, thus increasing its potency compared to wt*Sj*SULT.

**Table 2:** Comparison of Hydrogen Bond Interactions between wt*Sj*SULT and m*Sj*SULT with OXA: Occupancy and Geometric Parameters

Compounds	H-Acceptor	H-Donor	Occupancy (%)	Distance (Å)	Angle (^o^)
Peroxisome proliferator-activated receptor delta/beta					
*Sm*SULT_OXA	ASP91-OD1	LIGAND-O3	54.87	2.62	167
	ASP144-OD2	LIGAND-N3	50.83	2.71	150
	ASP91-OD2	LIGAND-O3	44.53	2.63	167
	ASP144-OD1	LIGAND-N3	17.77	2.69	160
	ASP144-OD1	LIGAND-N3	8.73	2.75	151
wt*Sj*SULT-OXA	LIGAND-O1	SER156-OG	8.53	2.82	158
	SER156-OG	LIGAND-O1	8.43	2.83	157
	LIGAND-N3	TYR39-OH	3.10	2.80	167
	LIGAND-N3	ASN140-ND2	2.60	2.92	163
	LIGAND-O1	ASN153-ND2	2.57	2.911	163
	ASP87-OD1	LIGAND-O1	1.87	2.75	165
	ASN153-O	LIGAND-O1	1.20	2.86	151
m*Sj*SULT-OXA	ASN140-OD1	LIGAND-N3	9.35	2.74	160
	ASN153-OD1	LIGAND-O1	9.31	2.73	163

### Structural Dynamics and Conformational Changes of *Sm*SULT and *Sj*SULT Variants during MD Simulations: Insights from RMSF and PCA Analysis

**Fig. 9:**
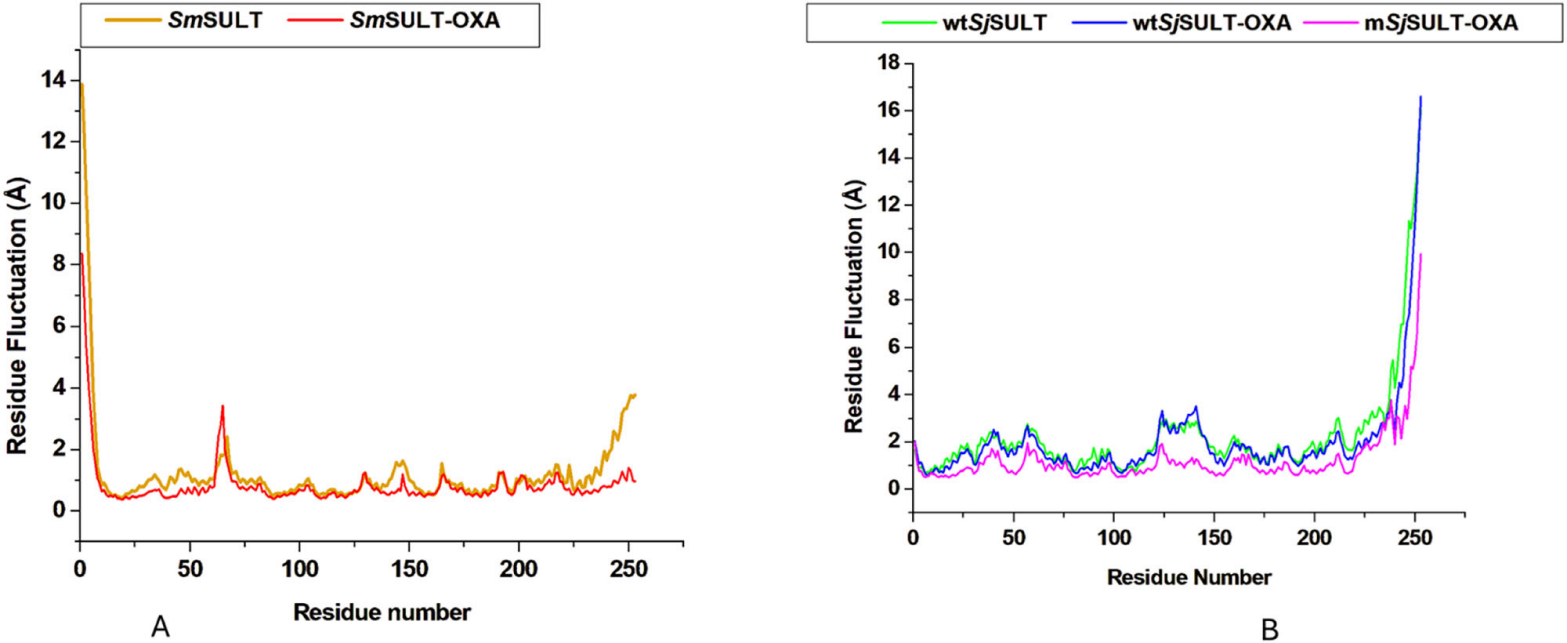
Residual Fluctuation of the SULT Variants Across the Entire Simulation. **A** Fluctuations in *Sm*SULT systems and **B** Fluctuations in *Sj*SULT systems

The RMSF analysis provides a measure of the flexibility of protein residues, reflecting how much each residue fluctuates around its average position during the simulation [37]. In the case of *Sm*SULT, the binding of OXA leads to a significant reduction in RMSF values, indicating increased rigidity and stability of the protein. The average RMSF for *Sm*SULT-OXA (0.83 Å) is notably lower than that of the unbound *Sm*SULT (1.21 Å) (Table 3), suggesting that OXA binding effectively stabilises the enzyme, enhancing its functional efficacy.. This is visually supported by the RMSF plot, where the *Sm*SULT-OXA complex consistently shows lower fluctuations across most residues compared to the unbound *Sm*SULT, particularly in the regions around residues 40–60 and 250–260, where the majority of the enzyme’s active site residues are located (Fig. 9A).

**Table 3:** Mean RMSD, RoG, and RMSF values (Å) for the Global Protein Structure, Active Site Residues, and Ligand in *Sm*SULT, *Sm*SULT-OXA, wt*Sj*SULT, wt*Sj*SULT-OXA, and m*Sj*SULT-OXA Complexes - Corresponding Replica-specific and Averaged Values from two Independent Simulations are Provided in Supplementary Table 2

Mean RMSD Values (Å)					
Structural Components	*Sm*SULT	*Sm*SULT-OXA	wt*Sj*SULT	wt*Sj*SULT-OXA	m*Sj*SULT-OXA
Global Protein Structure	2.69 ± 0.54	1.41 ± 0.49	7.79 ± 1.37	3.24 ± 0.89	4.48 ± 0.53
Active Site Residues	2.1 ± 0.49	1.29 ± 0.13	2.11 ± 0.36	2.69 ± 0.37	2.26 ± 0.27
Ligand		1.75 ± 0.38	1.6 ± 0.27	1.05 ± 0.32	
Mean RoG Values (Å)					
Global Protein Structure	18.44 ± 0.25	18.14 ± 0.08	18.54 ± 0.44	19.35 ± 0.33	18.56 ± 0.21
Active Site Residues	9.59 ± 0.22	9.31 ± 0.06	9.5 ± 0.17	9.81 ± 0.18	9.42 ± 0.52
Ligand		3.93 ± 0.08		3.86 ± 0.09	3.98 ± 0.05
Mean RMSF Values (Å)					
Global Protein Structure	1.21 ± 0.1	0.83 ± 0.02	2.24 ± 0.18	1.98 ± 0.03	1.20 ± 0.01
Binding Site Residues	0.89 ± 0.03	0.52 ± 0.09	2.02 ± 0.06	1.79 ± 0.07	1 ± 0.04

In the case of wt*Sj*SULT, the presence of OXA does not significantly stabilise the protein, as reflected by the RMSF values. The RMSF plot shows that wt*Sj*SULT-OXA (1.98 Å) has fluctuations closely comparable to the unbound wt*Sj*SULT (2.24 Å) (Table 3). Notably, significant fluctuations are observed in the regions around residues 100–150 and 250–270, which could explain its reduced effectiveness due to less stabilisation in these critical regions upon OXA binding (Fig. 9B). However, in the m*Sj*SULT variant, the average RMSF decreases substantially, indicating a more rigid and stabilised structure upon OXA binding. The RMSF for m*Sj*SULT-OXA (1.2 Å) is comparable to that observed for *Sm*SULT-OXA, suggesting that these mutations have restored the ability of OXA to induce a conformational state similar to that in *Sm*SULT (Table 3). The RMSF plot clearly shows this reduction in fluctuation, particularly in the same critical regions (100–150 and 250–270), where the m*Sj*SULT-OXA complex maintains lower residue flexibility across most of the protein, closely mirroring the stabilisation pattern seen in *Sm*SULT-OXA.

**Fig. 10:**
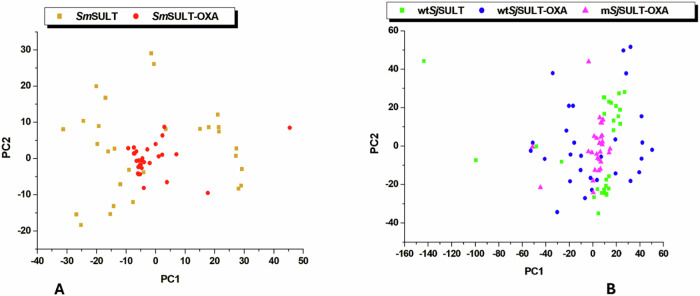
Principal component analysis of **A** *Sm*SULT systems and **B** *Sj*SULT systems

The PCA analysis further substantiates the observations drawn from the RMSF data, offering deeper insights into the conformational dynamics of the studied systems [38]. By projecting protein mobility into two principal components, PCA effectively captures the most significant patterns of structural variability. The first principal component (PC1) accounts for the greatest variance in the protein’s conformational states, while the second principal component (PC2) highlights the next most significant variance [39]. For *Sm*SULT, the PCA plot illustrates a clear distinction between the unbound protein and the *Sm*SULT-OXA complex. The unbound *Sm*SULT displays greater dispersion across both PC1 and PC2, indicating a broader range of conformational states (Fig. 10). This suggests that, in the absence of OXA, *Sm*SULT explores a more diverse set of structural configurations. In contrast, the *Sm*SULT-OXA complex exhibits a more converged distribution in the PCA plot, signifying a more restricted conformational space when OXA is bound. This convergence is indicative of the stabilising effect that OXA has on *Sm*SULT, corroborating the lower RMSF values observed in the RMSF analysis.

In the case of *Sj*SULT, the PCA analysis reveals a more nuanced pattern. The wt*Sj*SULT system, particularly when bound to OXA, is predominantly dispersed along PC1, reflecting considerable conformational flexibility. This flexibility aligns with the higher RMSF values observed for wt*Sj*SULT-OXA, suggesting that the binding of OXA does not significantly stabilise the wild-type protein structure. However, the m*Sj*SULT-OXA complex shows a much more converged distribution in the PCA plot, similar to the pattern observed in *Sm*SULT-OXA (Fig. 10). This convergence suggests that the mutations in m*Sj*SULT lead to a more defined and less variable conformational landscape, effectively enhancing the structural stabilisation of the protein when OXA is bound.

The contrast between the PCA plots of wt*Sj*SULT-OXA and m*Sj*SULT-OXA is particularly telling. While the former displays greater dispersion, indicating a wide range of conformations, the latter shows a tight clustering of data points. This clustering reflects a more restricted and stabilised conformational space, similar to that observed in *Sm*SULT-OXA. The parallels between the PCA plots of *Sm*SULT-OXA and m*Sj*SULT-OXA suggest that the mutations in m*Sj*SULT have restored the ability of OXA to induce a conformational state that is more effective for its function. Moreover, the convergence observed in the PCA plot of m*Sj*SULT-OXA, particularly along PC1, indicates that this component is highly correlated with the most significant conformational shifts in the protein. These shifts are likely crucial for the protein’s functional dynamics, as they represent the primary mode of motion that influences the protein’s interactions and overall activity [40]. While PC2 captures an important, though secondary, mode of motion, PC1 remains the primary focus in PCA because of its ability to reveal the dominant patterns of protein movement that are most relevant to the protein’s function and its interaction with OXA [40].

### Assessing the Stability of Protein Configurations in *Sm*SULT and *Sj*SULT with OXA Binding

**Fig. 11:**
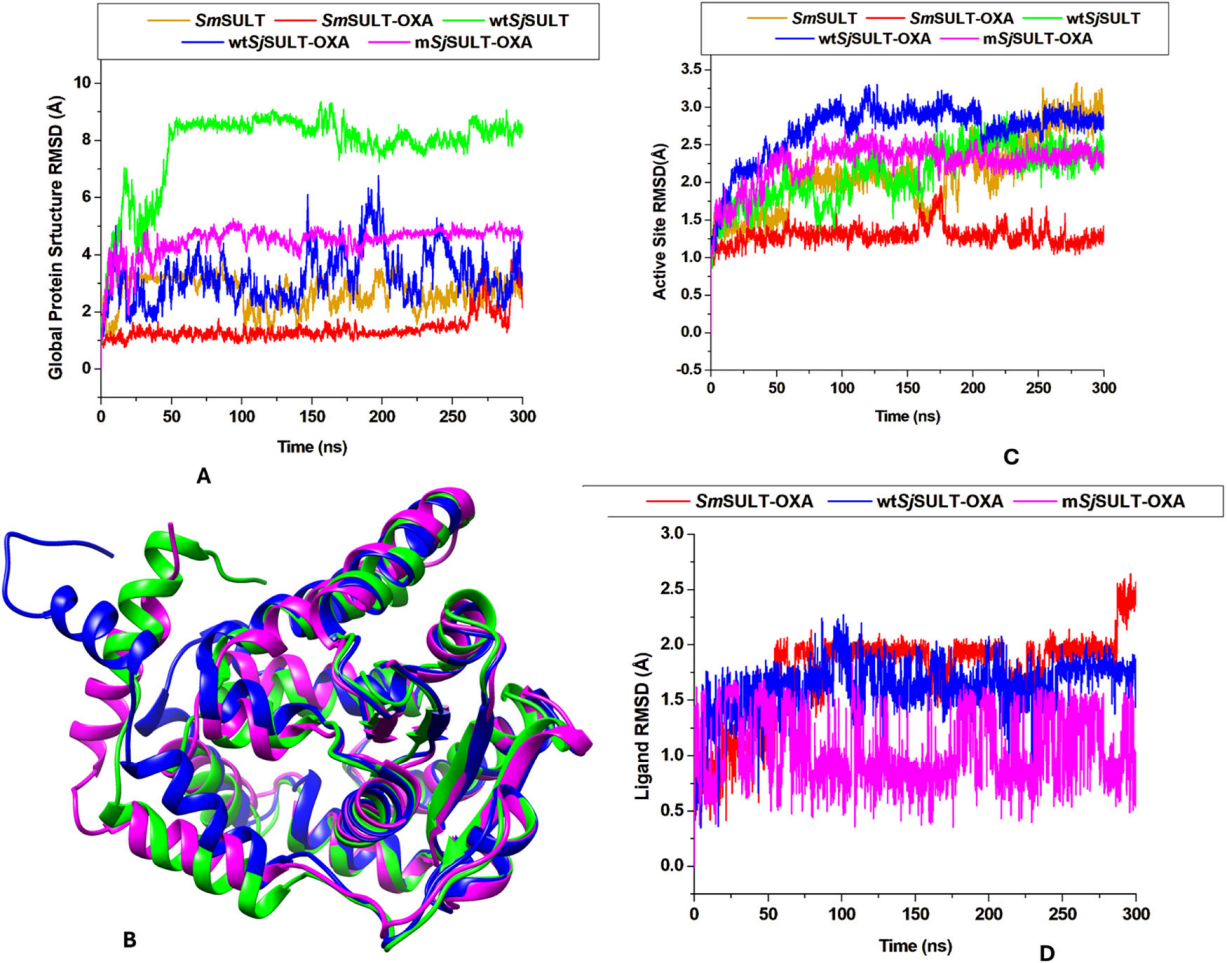
Pictorial representation of the RMSD Patterns in **A** The global structures of *Schistosoma* SULT systems. **B** Superposition of wt*Sj*SULT (green), wt*Sj*SULT-OXA (blue), and m*Sj*SULT-OXA (magenta) at 100 ns. **C** RMSD patterns of the active sites, and **D** Ligand stability in the *Schistosoma* SULT systems

The RMSD analysis offers valuable insights into the stability and conformational changes of protein structures under various conditions [41]. The RMSD trajectory plot reveals that the binding of OXA to *Sm*SULT leads to a significant stabilisation of the protein’s conformation throughout the MD simulation. This is evidenced by the consistently horizontal trajectory of the *Sm*SULT-OXA complex, which remains below that of the unbound *Sm*SULT (Fig. 11A). The lower average RMSD for *Sm*SULT-OXA, at 2.69 Å, indicates minimal deviation from the initial structure, signifying a more stable conformation upon OXA binding. In contrast, the RMSD trajectory of unbound wt*Sj*SULT (Apo) displays a marked upward deviation, with an average RMSD of 7.79 Å, reflecting significant conformational changes and instability (Table 3).

When comparing the RMSD trajectories of the bound *Sj*SULT variants, m*Sj*SULT-OXA demonstrates a more consistent and stable structure relative to wt*Sj*SULT-OXA, which exhibits irregular fluctuations. The average RMSD for wt*Sj*SULT-OXA is 3.24 Å, whereas m*Sj*SULT-OXA has a slightly higher average RMSD of 4.48 Å (Table 3). Although m*Sj*SULT-OXA has a marginally higher RMSD than wt*Sj*SULT-OXA, the plot shows that the binding of OXA to m*Sj*SULT modulates its stability throughout the simulation. In contrast, wt*Sj*SULT-OXA, despite its lower RMSD, displays erratic stability trends, indicating that m*Sj*SULT-OXA achieves a more controlled and consistent structural conformation upon OXA binding (Fig. 11A, B).

Examining the active site RMSD reveals patterns consistent with the global RMSD observations (Fig. 11C). For *Sm*SULT and *Sm*SULT-OXA, the active site RMSD trajectories reflect the overall stability, with average values of 2.1 and 1.29 Å, respectively (Table 3). These values further confirm the stabilising effect of OXA on the *Sm*SULT active site. Conversely, a more complex pattern emerges in the *Sj*SULT systems. The active site RMSD for wt*Sj*SULT-OXA remains higher compared to other variants, with an average RMSD of 2.69 Å. In contrast, m*Sj*SULT-OXA exhibits a lower average active site RMSD of 2.26 Å, suggesting that the mutations in m*Sj*SULT enhance the stability of the active site upon OXA binding compared to wt*Sj*SULT (Table 3).

Finally, the ligand RMSD plot illustrates the dynamics of OXA within the binding pocket of each protein variant. The trajectory for m*Sj*SULT-OXA shows the lowest ligand RMSD, averaging 1.05 Å, despite some observed movement. This suggests that OXA undergoes conformational adjustments within the m*Sj*SULT binding pocket to optimise its interactions with the active site residues (Table 3). In contrast, the ligand RMSD for *Sm*SULT-OXA and wt*Sj*SULT-OXA reveals similar patterns, with average values of 1.75 and 1.6 Å, respectively. These findings indicate that m*Sj*SULT-OXA exhibits the most stable OXA binding, implying that the mutations introduced in m*Sj*SULT enhance the protein’s binding affinity and overall stability, making it the most effective binder among the *Sj*SULT variants. Results from duplicate simulations revealed consistent trends across all systems. Replica-specific plots for global RMSD, active site dynamics, ligand stability, and residue fluctuations are provided in Supplementary Figs S1–S5.

### Assessment of Protein and Ligand Stability through Radius of Gyration Analysis

**Fig. 12:**
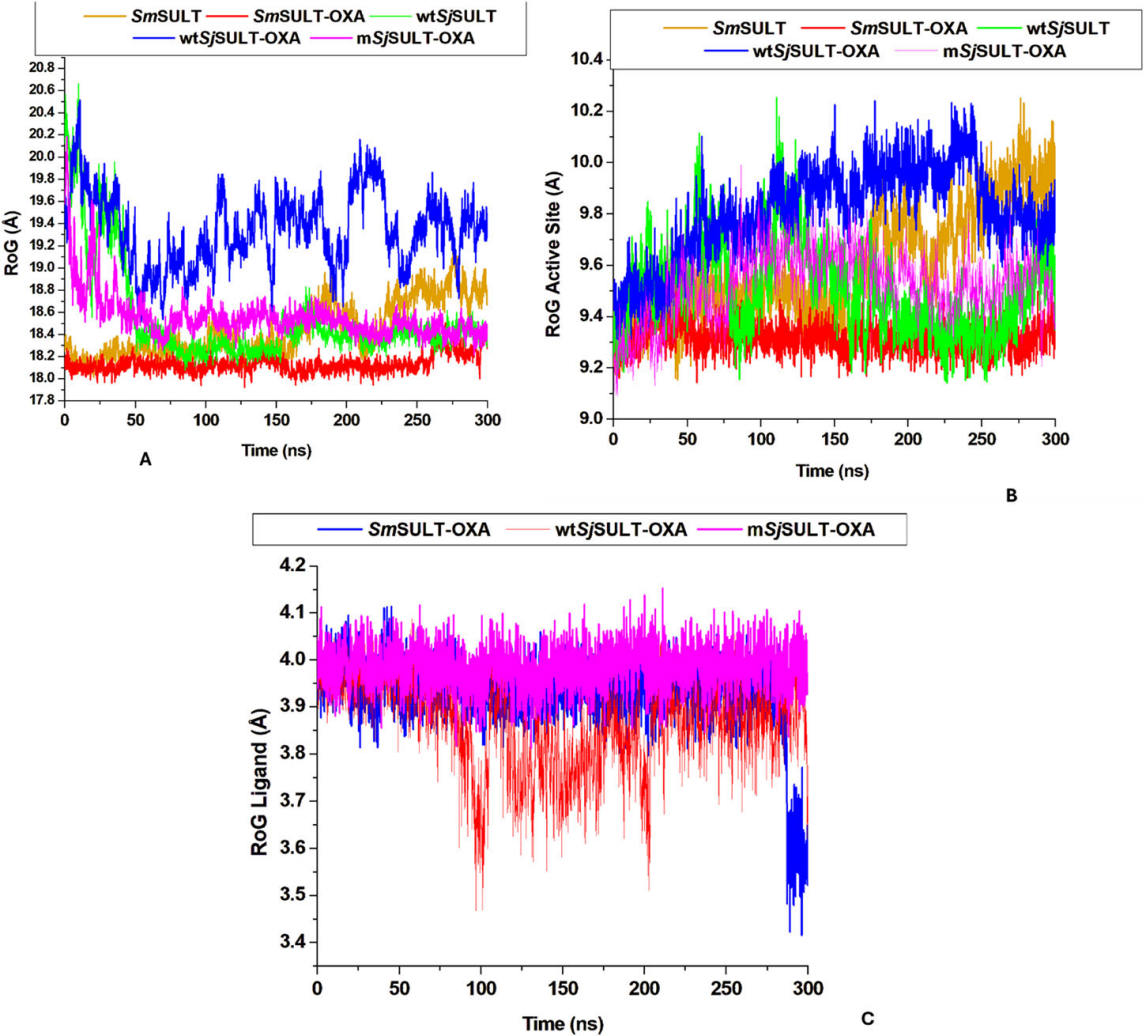
Pictorial representation of the RoG patterns in **A** The global structures of *Schistosoma* SULT systems, **B** Their active sites, and **C** Ligand stability in the *Schistosoma* SULT systems

The Radius of Gyration (RoG) analysis provides valuable insights into the structural changes induced by ligand binding across different SULT variants. The average RoG values reveal key differences in how OXA affects the compactness and stability of the protein structures. For *Sm*SULT, the RoG decreases from 18.44–18.14 Å upon OXA binding, indicating a more compact and stable conformation when the ligand is present (Table 3). This reduction in RoG signifies enhanced structural stability, aligning with the observed increase in efficacy for *Sm*SULT in the presence of OXA. The compact conformation likely facilitates more effective ligand interaction, contributing to the higher efficacy of OXA in *Sm*SULT (Fig. 12A). In contrast, *Sj*SULT shows a different pattern. The average RoG values are 18.54 Å for unbound *Sj*SULT, 19.35 Å for wt*Sj*SULT-OXA, and 18.56 Å for m*Sj*SULT-OXA. The slight increase in wt*Sj*SULT-OXA compared to unbound *Sj*SULT suggests that OXA binding induces less efficient stabilization in wt*Sj*SULT-OXA, which may explain why OXA is less effective in this variant.

The active site RoG values further validate the impact of OXA binding on protein structure. For *Sm*SULT, the active site RoG decreases from 9.59–9.31 Å upon binding with OXA, demonstrating a notable increase in the compactness and stability of the active site. This reduction in RoG reflects a more stable and compact conformation, aligning with the enhanced efficacy observed for *Sm*SULT. On the other hand, for wt*Sj*SULT-OXA and m*Sj*SULT-OXA, the active site RoG values are 9.81 and 9.42 Å, respectively (Table 3). The higher active site RoG for wt*Sj*SULT-OXA suggests that OXA does not stabilize the active site as effectively in this variant compared to *Sm*SULT, contributing to its lower efficacy. However, m*Sj*SULT-OXA displays an active site RoG of 9.42 Å, which mirrors the trend seen in *Sm*SULT-OXA, indicating that OXA induces a similar increase in compactness and stability. These findings highlight that while OXA binding enhances stability and compactness at the active site in both *Sm*SULT and m*Sj*SULT, it does so more effectively in *Sm*SULT compared to wt*Sj*SULT (Fig. 12B).

The Radius of Gyration (RoG) trajectories for the ligands across the different systems display similar patterns, with overlapping plots making detailed movement analysis challenging (Fig. 12C). The average ligand RoG values are 3.93 Å for *Sm*SULT-OXA, 3.86 Å for wt*Sj*SULT-OXA, and 3.98 Å for m*Sj*SULT-OXA. Interestingly, despite the lower efficacy observed with wt*Sj*SULT, the ligand appears to be more stable in this system compared to the others, as indicated by these average values (Table 3).

### OXA-induced Perturbation of Protein Hydrophobicity and Solvent Accessibility

The overall changes in OXA binding and residue interactions were quantified using Solvent Accessible Surface Area (SASA). This metric assesses the mobility and exposure of residues to the solvent, providing insights into the perturbations within different protein and ligand regions [42]. High SASA values indicate decreased hydrophobicity, while lower SASA values suggest increased hydrophobicity [42]. Specifically, *Sm*SULT-OXA’s trajectory indicates that OXA performs optimally in a hydrophobic setting, aligning with enhanced functional activity. In the *Sj*SULT systems, m*Sj*SULT-OXA shows the lowest SASA, indicating it is the most hydrophobic and thus exhibits superior functional activity compared to wt*Sj*SULT-OXA. The estimated average SASA values for the global structure are 11917 ± 301.43 for *Sm*SULT and 11264.27A^2^ for *Sm*SULT-OXA (Table 4). In comparison, wt*Sj*SULT, wt*Sj*SULT-OXA, and m*Sj*SULT-OXA have average SASA values of 14040.23A^2^, 14218.15A^2^, and 13475.09A^2^, respectively. This increase in hydrophobicity likely accounts for the potency of OXA in both proteins.

**Table 4:** Mean SASA Values (A^2^) for the Global Protein Structure, Active Site Residues, and Ligand in *Sm*SULT, *Sm*SULT-OXA, wt*Sj*SULT, wt*Sj*SULT-OXA, and m*Sj*SULT-OXA Complexes

Mean SASA Values (A^2^)					
Structural Components	*Sm*SULT	*Sm*SULT-OXA	wt*Sj*SULT	wt*Sj*SULT-OXA	m*Sj*SULT-OXA
Global Protein Structure	11917 ± 301.43	11264.27 ± 281.91	14040.23 ± 317.33	14218.15 ± 293.46	13475.09 ± 265.28
Active Site Residues	876.53 ± 123.06	289.64 ± 54.95	1075.79 ± 88.15	903.04 ± 93.2	639.59 ± 56.44
Ligand		38 ± 11.51		106.39 ± 20.66	76.04 ± 16.39

**Fig. 13:**
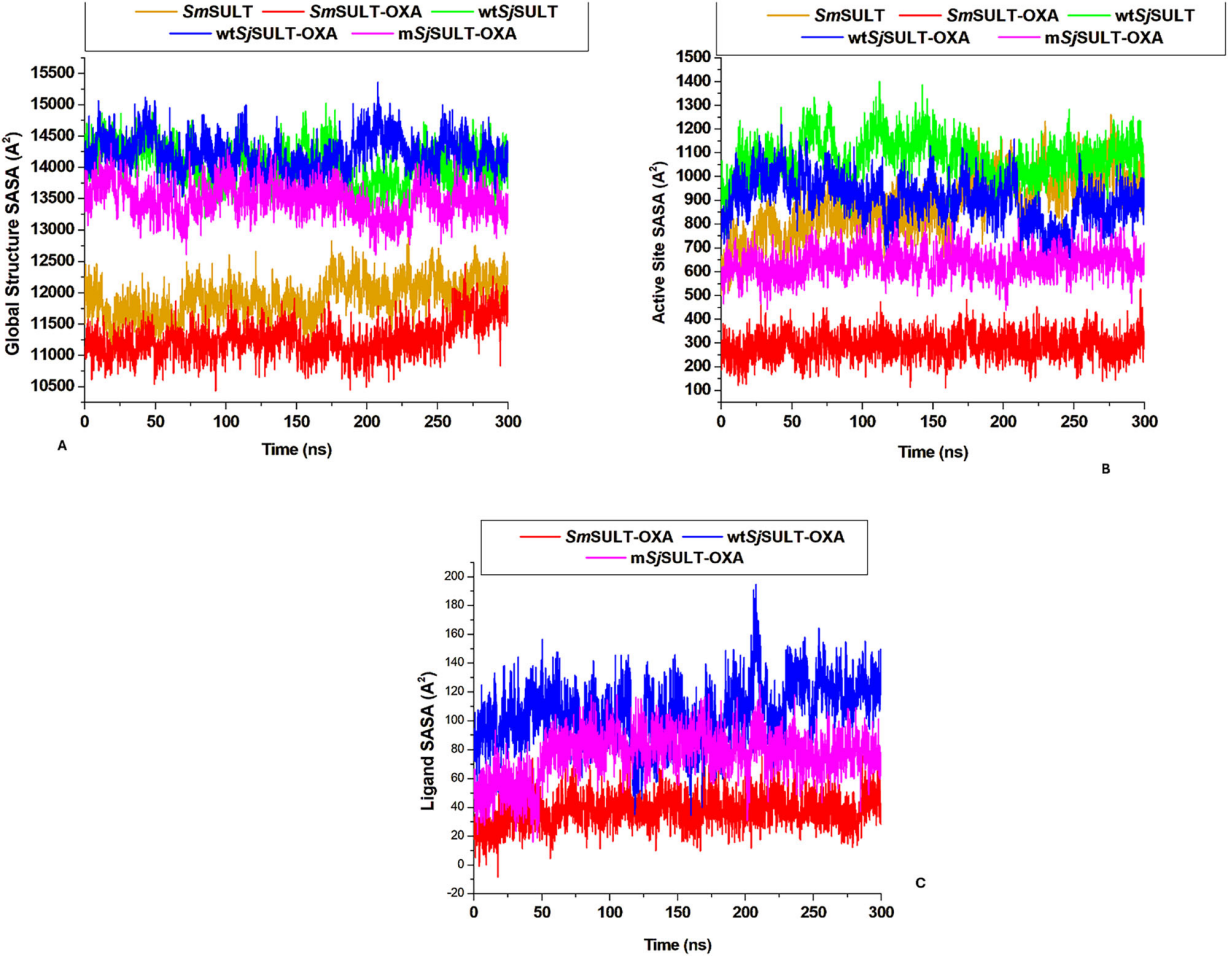
Pictorial representation of the Solvent Accessible Surface Area patterns in **A** The global structures of *Schistosoma* SULT systems, **B** Their active sites, and **C** Ligand stability in the *Schistosoma* SULT systems

Analysis of the active site residue SASA reveals that *Sm*SULT-OXA exhibits the lowest value, consistent with the global structure SASA results, followed by m*Sj*SULT-OXA. This suggests that OXA binding promotes a shift of surface-exposed residues towards the hydrophobic core, enhancing optimal binding interactions (Fig. 13B). The ligand SASA for *Sm*SULT and *Sm*SULT-OXA shows a consistent, horizontal movement throughout the MD simulation, suggesting that OXA remains well-buried within the binding pocket of both *Sm*SULT and *Sm*SULT-OXA (Fig. 13C). Conversely, the wt*Sj*SULT-OXA plot shows noticeable irregularities, indicating less stable binding of OXA in the pocket compared to *Sm*SULT-OXA, where OXA is more consistently accommodated (Fig. 13C).

## Conclusion

This study successfully elucidates the structural mechanisms underlying the selective efficacy of OXA against *Schistosoma* species. Our comprehensive molecular simulations reveal that OXA binding leads to significant conformational changes in *Sm*SULT, resulting in enhanced stability and high enzymatic activity, which aligns with its observed effectiveness. Conversely, OXA binding to wt*Sj*SULT does not induce similar stabilisation, which explains its lower efficacy. The mutation from *Val139* to *Gly139* in *Sj*SULT effectively restores OXA’s binding affinity and activity, highlighting the critical role of specific amino acid residues in ligand activation.

Unlike prior structural studies limited to static snapshots, our approach captures the dynamic behaviour of the enzyme–ligand complex, enabling a more nuanced understanding of selective efficacy. These findings confirm the hypothesis that structural variations and residue-specific interactions are key determinants of OXA’s selective efficacy. Overall, our study provides valuable insights into the molecular basis of OXA’s activity, contributing to a deeper understanding of its selective potency and offering potential avenues for targeted therapeutic interventions against *Schistosoma* infections.

## Supplementary information

Supplementary File

## Data Availability

No datasets were generated or analysed during the current study.
