# Supplementary material for: Structural Mechanisms Driving the Selective Efficacy of Oxamniquine against Schistosoma mansoni and Schistosoma japonicum
Source: Cell Biochem Biophys. 2025 Apr 19;83(3):3815–31. doi: 10.1007/s12013-025-01756-9 (PMC12414012; doi:10.1007/s12013-025-01756-9)
Supplement: Supplementary file 1 — Supplementary File [file 12013_2025_1756_MOESM1_ESM.docx]

**Structural Mechanisms Driving the Selective Efficacy of Oxamniquine Against *Schistosoma* *mansoni* and *Schistosoma japonicum*.**

**Supplementary File**

This file contains replica analyses supporting the molecular dynamics simulations presented in the main manuscript. Figures S1–S5 and Tables S1–S3 provide comparative results from duplicate simulations, demonstrating the reproducibility of key trends observed in global RMSD, active site stability, ligand behavior, residue flexibility, and binding free energy estimations. Plots, graphs, and tables presented in the main manuscript are derived from Replica 1, while Replica 2 results are presented here for comparison and validation.

.



**Replica 1**

**

**

**Replica 2**

**Figure S1:** Comparison of global RMSD patterns between Replica 1 and Replica 2 in (A) *Sm*SULT, (B) *Sm*SULT-OXA, (C) wt*Sj*SULT, (D) wt*Sj*SULT-OXA, and (E) m*Sj*SULT-OXA over the 300 ns MD trajectory.


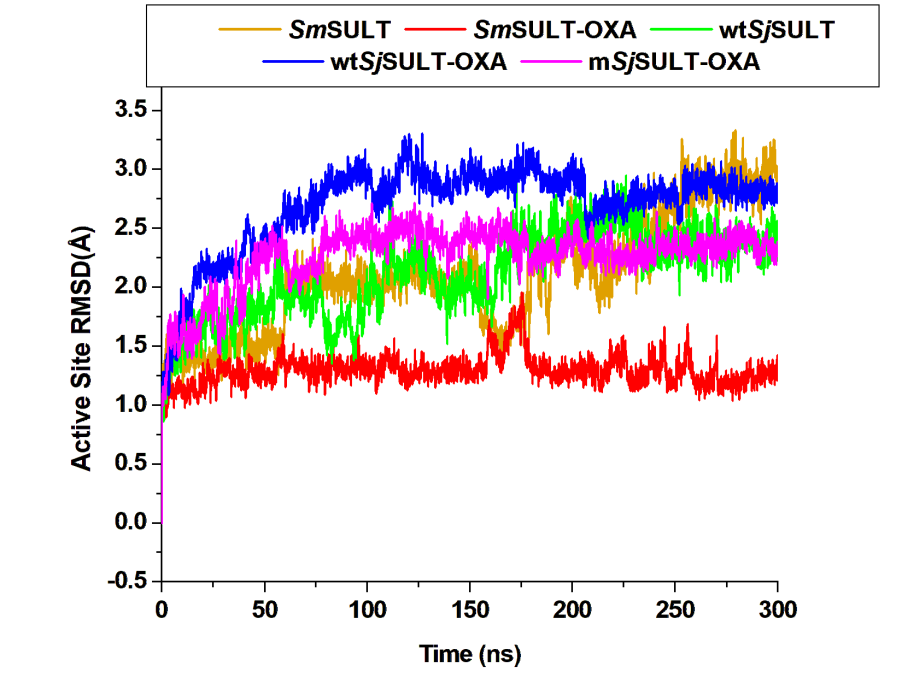


**Replica 1**





**Replica 2**

**Figure S2:** Overlay of RMSD trajectories of active site residues in (A) *Sm*SULT, (B) *Sm*SULT-OXA, (C) wt*Sj*SULT, (D) wt*Sj*SULT-OXA, and (E) m*Sj*SULT-OXA in Replica 1 and Replica 2.





**Replica 1**





**Replica 2**

**Figure S3:** Stability of the OXA ligand throughout the 300 ns MD simulation in each SULT complex, comparing Replica 1 and Replica 2.





**Replica 1**





**Replica 2**

**Figure S4**: Residue-level fluctuations in (A) *Sm*SULT and (B) *Sm*SULT-OXA showing consistent flexibility patterns across Replica 1 and Replica 2.





**Replica 1**





**Replica 2**

**Figure S5:** RMSF plots for (A) wt*Sj*SULT, (B) wt*Sj*SULT-OXA, and (C) m*Sj*SULT-OXA illustrating structural flexibility across both replicas.

**Table 1: Energy components (kcal/mol) for the interaction of OXA with *Sm*SULT, wt*Sj*SULT, and m*Sj*SULT complexes across two independent simulation**

|  | **Energy Components (kcal/mol)** | | | | | | |
| --- | --- | --- | --- | --- | --- | --- | --- |
| **Complexes** | **∆E_vdW_** | **∆E_ele_** | **∆G_gas_** | **∆G_ele,sol(GB)_** | **∆G_np,sol_** | **∆G_sol_** | **∆G_bind_** |
| ***Sm*SULT-OXA**  **(Replica 1)** | -39.83±0.13 | -294.97±0.49 | -334.41±0.48 | 292.27±0.42 | -5.90±0.098 | 286.37±0.41 | -48.04±0.17 |
| ***Sm*SULT-OXA**  **(Replica 2)** | -38.55±0.17 | -291.28±0.61 | -329.83±0.62 | 288.69±0.52 | -5.80±0.012 | 282.88±0.52 | -46.94±0.23 |
| ***Sm*SULT-OXA**  **Mean** | -39.19 | -293.13 | -332.12 | 290.48 | -5.85 | 284.63 | 47.49 |
| **wt*Sj*SULT-OXA**  **(Replica 1)** | -33.6±0.15 | 77.06±0.84 | 43.47±0.85 | -61.61±0.8 | -4.7±0.02 | -66.31±0.79 | -22.84±0.23 |
| **wt*Sj*SULT-OXA**  **(Replica 2)** | -35.92±0.16 | 84.63±0.89 | 48.71±0.93 | -66.36±0.8 | -5.01±0.02 | -71.44±0.80 | -22.72±0.25 |
| **wt*Sj*SULT-OXA**  **Mean** | -34.76 | 80.84 | 46.09 | -63.99 | -4.86 | -68.89 | -22.78 |
| **m*Sj*SULT-OXA**  **(Replica 1)** | -40.02±0.16 | -144.23±0.54 | -184.26±0.56 | 150.49±0.54 | -5.47±0.01 | 145.02±0.53 | -39.23±0.15 |
| **m*Sj*SULT-OXA**  **(Replica 2)** | -32.87±0.18 | -129.11±0.90 | -161.98±0.97 | 136.82±0.79 | -4.6±0.02 | 132.21±0.78 | -42.77±0.28 |
| **m*Sj*SULT-OXA**  **Mean** | -36.46 | -136.67 | -173.12 | 143.66 | -5.01 | 138.62 | 41.00 |

1. **Table 2: Mean RMSD, RoG, and RMSF values (Å) for the global protein structure, active site residues, and ligand in *Sm*SULT, *Sm*SULT-OXA, wt*Sj*SULT, wt*Sj*SULT-OXA, and m*Sj*SULT-OXA complexes**

| **Mean RMSD Values (Å)** | | | | |  | |
| --- | --- | --- | --- | --- | --- | --- |
| **Structural**  **Components** | ***Sm*SULT** | ***Sm*SULT-OXA** | **wt*Sj*SULT** | **wt*Sj*SULT-OXA** | **m*Sj*SULT-OXA** |  |
| Global Protein Structure (Replica 1) | 2.69±0.54 | 1.41±0.49 | 7.79±1.37 | 3.24±0.89 | 4.48±0.53 |  |
| Global Protein Structure (Replica 2) | 2.61±0.01 | 2.20±0.014 | 5.27±0.03 | 3.14±0.02 | 4.47±0.03 |  |
| Mean | 2.65 | 1.81 | 6.53 | 3.19 | 4.48 |  |
| Active  Site Residues (Replica 1) | 2.1±0.49 | 1.29±0.13 | 2.11±0.36 | 2.69±0.37 | 2.26±0.27 |  |
| Active  Site Residues (Replica 2) | 2.05±0.04 | 1.59±0.25 | 2.29±0.29 | 3.13±0.44 | 2.21±0.43 |  |
| Mean | 2.08 | 1.44 | 2.2 | 2.91 | 2.23 |  |
| Ligand RMSD (Replica 1) |  | 1.75±0.38 | 1.6±0.27 | 1.05±0.32 |  |  |
| Ligand RMSD (Replica 2) |  | 1.77±0.15 | 1.47±0.24 | 1.25±0.37 |  |  |
| Mean |  | 1.76 | 1.53 | 1.15 |  |  |
| **Mean RMSF Values (Å)** | | | | |  | |
| Global Protein  Structure (Replica 1) | 1.21±0.1 | 0.83±0.02 | 2.24±0.18 | 1.98±0.03 | 1.20±0.01 |  |
| Global Protein  Structure (Replica 2) | 1.18±0.09 | 0.99±0.07 | 2.06±0.11 | 1.88±0.14 | 1.73±0.11 |  |
| Mean | 1.20 | 0.91 | 2.15 | 1.93 | 1.47 |  |
| Binding  Site Residues (Replica 1) | 0.89±0.03 | 0.52±0.09 | 2.02±0.06 | 1.79±0.07 | 1±0.04 |  |
| Binding  Site Residues (Replica 2) | 0.79±0.03 | 0.98±0.07 | 1.87±0.14 | 1,38±0.06 | 1.48±0.19 |  |
| Mean | 0.84 | 0.75 | 1.94 | 1.59 | 1.24 |  |
